# The Medical Evidence of Crime

**Published:** 1863-04

**Authors:** 


### Art. XV.—THE MEDICAL EVIDENCE OF CRIME.

In the March number of the *Cornhill Magazine* there is a paper bearing the title which we have placed above—"The Medical Evidence of Crime." We are not ambitious to enter into controversy with our contemporaries; but there are, in the communication referred to, so many points touched upon which bear on the profession of medicine, that it would be impossible to avoid them in any effort to supply sound information to the public on the very important subject to which the *Cornhill* writer has invited attention.

The professional reader who shall consult the pages of the *Cornhill* will be led first to examine as to the source of the article named. He will not be so rudely inquisitive as to ask for names, but he will almost involuntarily put it to himself whether the composition is or is not the work of an expert, who has an interest in teaching the expert side of the question to the benighted masses.

But the query will not easily be answered, for the article may have one of two origins, each extreme the one from the other. It may be the work of a really artless man, who never gave scientific evidence in his life—who knows, in fact, as little of it as a home-bred Esquimaux knows of tropical botany; or, he may be a man who has made the delivery of evidence a pro found and profitable study, and who, to obtain a new hearing, has assumed, with considerable skill, that order of expression in which art is made to conceal art.

In the politest manner conceivable our *Cornhill* friend opens his attack by the usual and terrible insinuation that slow and secret poisoning is a practice at this time prevalent in England. He admits the absence of specific proofs, but fosters the speculations on which the "vague uneasiness" of the public rests. Thence he goes on to say that "an evil confessedly does exist," and quietly thrusts out the statement that for the last twenty-five years the English people have been unusually careless in affording protection to human life. There is, he suggests, constant failure of justice in cases of murder, even when a formal trial is opened; so that, in fact, if the writer is to be accepted as an authority, murderers, at the present day, are virtually under the patronage of the public, and, in so far as suspicion would carry a nervous man, are as numerous in the community as green peas in June. The ideal is presented with such delicacy of touch that it is barely discernible, and yet with such mysterious surroundings as to create a startling impression, sufficient to set every man in every family on the alert as to the murderer who is at his elbow, and to excite an universal terror amongst the afflicted who are obliged to seek, from relatives and friends, for that assistance which their own infirmities imperatively demand. Having chastised his reader to this agreeable acceptance of belief, our friend next turns to the philosophical, or as he would probably call it, the practical lesson. He commits himself to a text, and then divides his discourse into three parts.—Poisoning is a common practice: that is the text:—

1. Because the persons who suspect poisoning are slow to act.

2. Because (we are giving the actual words before us) "the whole course of English trials for poisoning seems specially designed to favour the escape of the criminal from justice."

3. Because the public is ill-informed as to the amount and kind of evidence which ought to be required to establish the fact of poisoning.

The three divisions thus given form the subjects to be afterwards discussed. Before we endeavour to determine their value, let us, however, inquire whether there is any truth in the suspicion that secret poisoning is one of the crafts of the present period.

To establish the presumption of this alleged fact, there is offered no line of what may be called direct evidence: but it is assumed that persons who make forensic inquiries their special study have certain private information respecting cases of poisoning that would give, if all the facts were out, such proof as must of necessity carry conviction. Now, if this be the case, it is too important a consideration to be allowed to be left for a single hour with doubts surrounding it. For our parts we do not quite see how such private knowledge can have been satisfactorily ascertained. If it rest on reliable data, then, as it seems to us, it must admit of ready and certain judicial investigation; nay, it ought, in common honesty, to be subjected to such investigation; if, on the other hand, it rest merely on suspicion—on suspicion not strong enough for it to be laid before a legal tribunal—then it is clear that there are no solid grounds for any accusation of the kind. Let us suppose a case in the way of elucidation. A man has been seized, we will assume, with symptoms of the choleraic type; a medical practitioner is called in, who treats the case as one of cholera: the patient dies, and the said medical man returns a certificate of death from cholera. As it appears to us, the case must either end here, in the burial of the dead man, or, suspicion being aroused, an inquest must be held, and the whole of the doubts must be clearly exposed, removed, or confirmed. After that inquiry, the doubt ceases, and the idea of secret poisoning is either demonstrated or destroyed: there is nothing left behind, in a word, that everybody does not know. On the contrary, if no inquiry were made, we can see no possibility of any expert obtaining knowledge behind the medical man who attended the case, or in the absence of a judicial inquiry, for which the services of the expert were summoned. Truly, such an occurrence might take place as this: a man might die; his death might be assigned by his medical man to a certain natural cause; and some suspicious friend, not content with the opinion, might ask an expert to go privately to work to ascertain whether the medical practitioner were right or wrong. But the conduction of an inquiry on these grounds involves so many dangers, that we cannot allow of its possibility. In the first place, a research of the kind would be an unprofessional act of which we can imagine no man guilty. But would it not also, if the inquiry elucidated death by foul means, place the investigator on the horns of a dilemma scarcely to be conceived? If in such an example he should proclaim his results, with what face would he meet his professional brethren? If he did not proclaim his results, with what face would he meet the world—he, the man who knew of the murder of another man, and held the secret solemnly in his own bosom? These are the alternatives, and with them before us we are bound to express that, in the absence of more light than has yet been thrown over the question, there is no reasonable evidence that any one has been poisoned whose death, returned by a qualified practitioner as from natural disease, remains uninvestigated and unknown except to a privileged few, who feel it advisable not to break the secret of murder.

Turning to the three positions to which the writer in the *Cornhill Magazine* has directed our attention, we dispute that medical men are in any degree improperly slow to act in cases where they suspect poison as a cause of disease. On the contrary, if the results of modern experience be taken into account, we have had presented in them a too hasty suspicion, and a nervous anxiety lest in the presence of doubtful symptoms foul play should have been overlooked. As we write, there is, in confirmation of this fact, a trial impending, in which a medical man is accused of having in a natural case of disease cast suspicions on a poor woman who is assumed, on the best evidence, to be innocent; and within the last few months we have known of another example in which a physician, by the merest accident, was prevented from exposing himself to the dangers of making a similar charge. In a word, the whole of the danger, both to the community and to the profession, lies in the tendency (unhappily set going by the popular clamour) of medical men to attach overweening doubts to symptoms which at the first blush appear unnatural, unaccountable, and unfamiliar. And, instead of hoping to see such tendency on the increase, it is for the welfare of the profession and the people that it should sink to its proper level. It is not for the medical man to close his eyes to the facts presented to him, nor for him to hold back for a moment from the expression of direct doubt in cases where he *really* sees that which is unnatural; but it is certainly his bounden duty to hold the strictest guard even over his fears, to be sure, and doubly sure, in his reasons for suspicion, to be deaf to all external influences having their origin in selfishness or ignorance, and to be guided by his own soundest judgment, before he breathe a word that shall afterwards be dubious or false. If medical men were for a moment to forget these principles (there is no fear that in the main they will), if they were to be moved by every passing sensation that happens, if they were to allow themselves to be the detectives instead of the doctors of those who give them their confidence, the end of the professional body would be at hand; we should be discarded in a body as false, lampooned as foolish, and cursed as cruel. The *Cornhill* writer ventures to tell medical men their duty in obscure cases of disease; who it is they should examine, who it is they should suspect, what material evidence they should gather together, what they should say, and what they should do. It is very suggestive and very kind of him to help the Esculapians on their weary way! He asks for the Chief Commissioner of Police "to be empowered by Act of Parliament to supply any medical man who may apply to him in a difficulty with the assistance of two medico-legal experts, paid servants of the Crown, and permanently appointed for this very purpose." He advises that these experts should attend suspected cases with the medical man, and suggest to him means of obtaining evidence as to the sources of poisoning. The theory is admirable; and we can imagine the satisfaction that an expert would feel in being entrusted by the Crown with the responsibilities of an office so concisely defined, with a salary attached to it commensurate with its dignity. But our friend talks without his host: he forgets that a private physician has no power to introduce a stranger into the house of his patient without permission; he ignores that, if the physician were permitted by law to enforce such entrance, the fact of his doing so would be equivalent to a charge of suspicion, which might be wrong; and he scrupulously avoids mentioning the circumstance that he has no such evidence of the existence of secret poisoning in England as would for a moment warrant so inquisitorial and absurd a project.

With equal speciousness or innocence the second proposition is argued—that in our Courts of Justice "the whole course of the proceeding seems specially designed to favour the escape of the criminal." When we reflect on the stern fact, that a criminal on trial for murder has no right of reply by his counsel; when we know in the majority of these cases the prosecution, backed by the Crown, has the means at its command to call into requisition the services of men who are assumed to be the most scientific and popular experts; when we see daily that in the way of scientific aid, the prisoner has to trust to voluntary effort; when we know that however innocent a man may be, the whole current of popular prejudice runs with the prosecution, and believes in its agents; we stand astounded to find a writer daring to say that everything is on the side of the accused. Whoever this statement-maker may be, whatever his object may be, we challenge him to take the trials for murder during the last ten years in England, and to show by the fees paid to scientific witnesses on the side of prosecution and defence, the value of the effort made by either party. We will undertake to say that on such a comparison, he would find for every ten pounds spent on the defence a hundred expended on the prosecution. It is, in fact, a common and well-known argument, that to give evidence for a prisoner is doubly insecure—"it is not respectable, and it does not pay;" to this argument, more than one man in our time who was morally innocent, has been ignorantly sacrificed. To prove the positive nature of the fact we have here recorded, there are two examples of immediate history before us. During the last circuit, two criminals, one a man named Fooks, another a youth named Burton, have been tried and convicted of wilful murder. There is not a shadow of a doubt that both these men were utterly irresponsible beings in respect to the crime which they committed. But were either of these unfortunates defended by proper scientific evidence? Neither. And why not? Because they had no means wherewith to meet the expenses of such aid. In respect to the boy Burton, his counsel, on the morning of the trial, in terms that were actually touching, beseeched the judge that some competent man should be summoned to testify to the jury the state of mind of his client. The judge refused, "because he had no funds at his disposal for the purpose," and thus the miserable victim of a well-known mental disorder was allowed to be condemned without proper defence, through sheer poverty. Had the *prosecution* in such a case wanted scientific evidence on its side, we know how readily the funds would have been forthcoming, and could have predicted who the men would have been who would have taken the funds for their evidence.

The statement made by the *Cornhill* writer on this question of defence of prisoners is, in a word, so solemnly and openly inconsistent with known facts, that it is with contempt we discuss it. If the person who put that statement to paper knows the truth, his act is a monstrous one; if he has spoken in ignorance, he is pitiable for daring to state on mere second hand or primed suggestion that which, by sober and honest inquiry, any man may disprove on positive evidence.

On the third point urged by the *Cornhill* writer, "that the public is ill-informed as to the amount and kind of evidence which ought to be required to establish the fact of poisoning," we agree with him; but with the inferences he draws we are as widely at issue. His reasoning is to the effect that in all cases the symptoms of felonious poisoning are to be distinguished from those of natural disease; and that, in fact, it is of small account whether or not chemical analysis detect poison. We do not deny that cases may occur where, on a person dying from the effects of a poison that has unmistakeably and specially its own symptoms, the cause of death may, by an intelligent man, be shrewdly predicated; and we acknowledge the acuteness of our opponent in adducing one solitary case of poisoning, by *aconite*, in support of his view; but we cannot with too much fervour condemn the sweeping dogmatic and dangerous deduction that is drawn from an isolated case where a moderately safe guess proved right. Let him take that of Dr. Smethurst, and try his hypothesis by that test. In that instance of forensic wisdom, the cause of the death of Miss Banks was attributed, in the first place, to arsenic, not, be it observed, on the pure symptoms, but on the chemical evidence that arsenic was found in the possession of the accused; and had that chemical evidence remained unaltered, he, the accused, would have been executed without the chance of a rescue. But it happened to be discovered before the trial that the man had no arsenic in his possession, and that the poison had crept in with the tests of the analyst. This important and basic error exposed, it was for the first time detected that the symptoms did not tally with the arsenic view in the pure and simple. Still the main question of a poison was sustained, and if our *Cornhill* advocate were right, the eminent men who investigated the symptoms should have agreed as to *the* nature of the poison. Were they? We analyse the evidence, and find that one remained true to *arsenic*, another moved to *antimony*, and adduced Strasbourg geese and *paté de foie gras* in proof of his interpretation: a third held for arsenic *or* antimony, but after the trial changed his mind, and wrote an elaborate letter to prove that the poison was mercury; a fourth, who was swayed at the trial in favour of arsenic, repented partly afterwards, and expressed that the symptoms were compatible with *natural disease;* while a fifth great man, who did more than give evidence, and who during the whole trial was certain that a *mineral* poison of some kind was used purposely to destroy the life of the poor woman, changed his tactics later in the day, and intimated his belief that Smethurst did not aim to kill Miss Banks, but wished to produce abortion, and for that end used a *vegetable* irritant that acted as the poison. Talk about the power of science dogmatically to separate natural disease from poison, and to name specific poisons of all kinds from symptoms, after such a Babel display as this! But the Smethurst trial is one only of its class.

It is argued that ignorance on these questions should be concealed: it is suggested that for the public safety it is best to have it believed that there are men so profound, that they can inevitably detect poison, whether the fact be so or not. We deny that either a suggested falsehood or a suppressed truth can ever be of service to any community. Second to none in our admiration of the advancing progress of our art, we affirm—because we will not be led by flat sophistry to state a falsehood—that there is no question on which we require so much light as the diagnosis of poisoning where the poison is not found; and we denounce as wicked the assumption that there are men who are so conversant with the positive phenomena of poisonings on the one hand, and the positive and unmistakeable courses of disease on the other, that they can lay down a law in every case on which a human life may be safely taken to satisfy the human ideal of required justice. Such pretended exactitude of knowledge is identical with the asseveration of the common quack, and the day will come when the public will see it in its true and clear light. We do not say these words to discourage honest inquirers from trying to make the diagnosis of poisons more accurate; we do not write from any humanitarian view in favour of criminals: for we feel for criminals as much as we do for those who, in the spirit of partizanship, are eager in the collection of evidence that shall tend to consign to death every man accused of crime; but we assert plainly the position of medicine in regard to her influence over a jury, and how far her assistance may be fairly estimated

The author of the paper to which we have adverted has naturally, after so completely elucidating his positions, thrown in a suggestion or two as to the way in which medical evidence of merit ought to be taken. We should not disagree widely with him on this point, nor object to a tribunal of experts to advise a jury; but to enter on these questions at this moment is not our object. There is matter for a special paper in the consideration of so vital a change in our modes of trial in criminal cases.

---

### Art. XVI.—ST. THOMAS'S AND BETHLEM HOSPITALS.

